# Algorithms for optimizing drug therapy

**DOI:** 10.1186/1472-6947-4-10

**Published:** 2004-07-20

**Authors:** Peter Wanger, Lene Martin

**Affiliations:** 1 Department of Clinical Neuroscience, Karolinska Institutet St Erik's Eye Hospital S-112 82 Stockholm Sweden

## Abstract

**Background:**

Drug therapy has become increasingly efficient, with more drugs available for treatment of an ever-growing number of conditions. Yet, drug use is reported to be sub optimal in several aspects, such as dosage, patient's adherence and outcome of therapy. The aim of the current study was to investigate the possibility to optimize drug therapy using computer programs, available on the Internet.

**Methods:**

One hundred and ten officially endorsed text documents, published between 1996 and 2004, containing guidelines for drug therapy in 246 disorders, were analyzed with regard to information about patient-, disease- and drug-related factors and relationships between these factors. This information was used to construct algorithms for identifying optimum treatment in each of the studied disorders. These algorithms were categorized in order to define as few models as possible that still could accommodate the identified factors and the relationships between them. The resulting program prototypes were implemented in HTML (user interface) and JavaScript (program logic).

**Results:**

Three types of algorithms were sufficient for the intended purpose. The simplest type is a list of factors, each of which implies that the particular patient should or should not receive treatment. This is adequate in situations where only one treatment exists. The second type, a more elaborate model, is required when treatment can by provided using drugs from different pharmacological classes and the selection of drug class is dependent on patient characteristics. An easily implemented set of if-then statements was able to manage the identified information in such instances. The third type was needed in the few situations where the selection and dosage of drugs were depending on the degree to which one or more patient-specific factors were present. In these cases the implementation of an established decision model based on fuzzy sets was required. Computer programs based on one of these three models could be constructed regarding all but one of the studied disorders. The single exception was depression, where reliable relationships between patient characteristics, drug classes and outcome of therapy remain to be defined.

**Conclusion:**

Algorithms for optimizing drug therapy can, with presumably rare exceptions, be developed for any disorder, using standard Internet programming methods.

## Background

During the last decades the possibility of computer support for optimum management of various disorders has attracted interest [1-3]. Regarding drug therapy the approach has usually been limited to one particular disease [4-7]. Diabetes has been extensively studied and thoroughly analyzed also from a systems perspective [8]. Several attempts have been made to develop a general model for medical guidelines, which could be used also for the development of computer programs [9-12]. Computerized physician order entry has been shown to reduce the frequency of serious medication errors. Decision support tools such as alerting functions for patient medication allergy are a key part of these applications. However, Abookire and co-workers [13] analyzed trend data obtained over a five-year period that showed decreasing compliance to allergy alert functions within computerized order entry. They concluded that optimal performance requires iterative refinement and that, as systems become increasingly complex, mechanisms to monitor their performance become increasingly critical [13]. In another study large differences were seen for all main types of medication errors: dose errors, frequency errors, route errors, substitution errors, and allergies [14]. In a university hospital setting, the reductions in transcription errors and medication turn-around times supported the view that computerized physician order entry and an electronic medication administration record can provide a good return on investment [15].

In the current study guidelines for 246 diseases were analyzed regarding the content of facts, and relationships between these facts, influencing the selection and dosage of therapeutic drugs. The aim of study was to identify one or more algorithms (descriptions of how to solve a problem in a definite number of steps), which could be used to support optimum drug therapy in any disease.

## Methods

The studied material consisted of 110 officially endorsed text documents, published between 1996 and 2004. Forty-four of these documents were published by the Swedish Medical Products Agency [16], 13 by the Swedish Agency for Evaluation of Medical Technologies [17] and 53 (in three booklets) by the Swedish Strategic Programme for The Rational Use of Antimicrobial Agents and Surveillance of Resistance (STRAMA) [18], established in order to prevent bacterial resistance to common antibiotics. The documents contained guidelines for drug therapy in altogether 246 disorders. Information regarding contraindications, dosage etc was available from an existing pharmaceutical website [19] and was retrieved by linking to context-relevant pages.

These documents were analyzed as follows: All statements in the particular document regarding patient-, disease- and drug-related factors were recorded. In a second step the statements in the texts describing the relationship between these factors were identified. After that the obtained information was rewritten in a format similar to a generic computer language, using if-then statements and logical operators as needed. These rewritten documents were grouped according to similarity in structure, based on the number of if-then statements, the need for branching or calculations in the code and the occurrence of 'left-overs', i.e. information in the text that could not be explicitly expressed using if-then statements and logical operators. In theory this procedure could result in an intractable combinatorial explosion. This was observed only in one situation, regarding side effects of anti-hypertensive medication.

## Results

**Figure 1:**
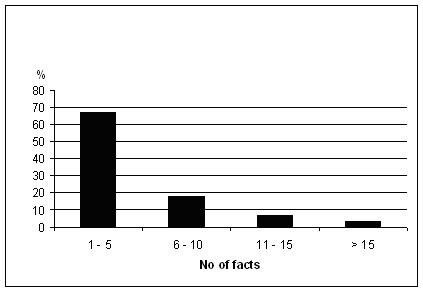
Number of facts influencing the selection of therapeutic drugs in the studied disorders (n = 246).

**Figure 2:**
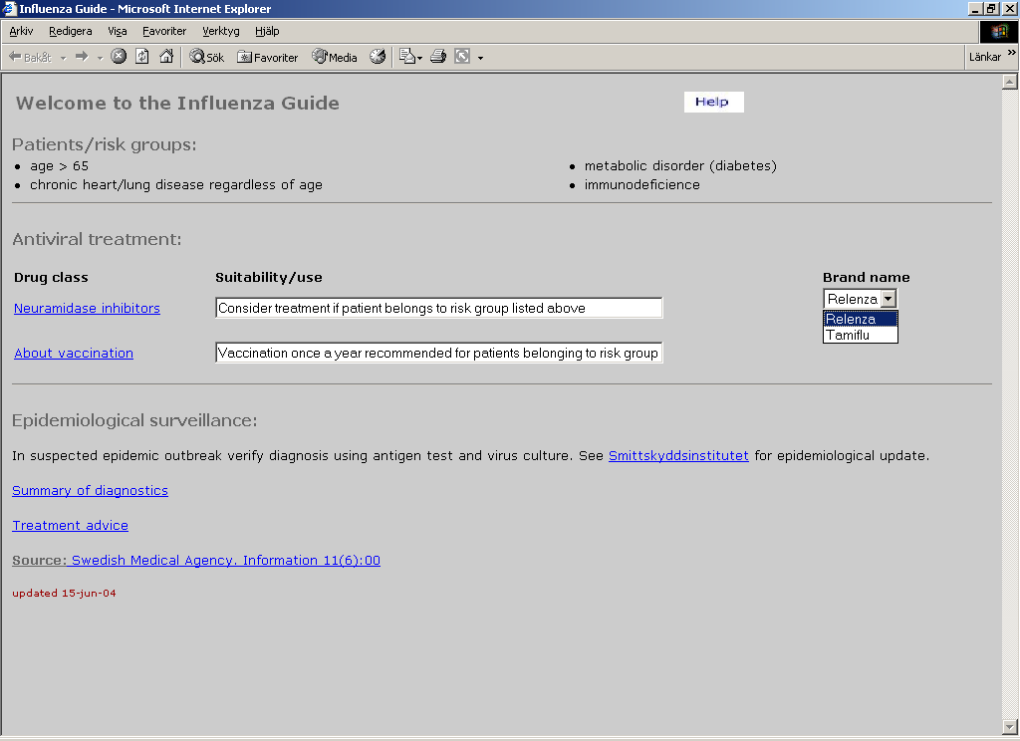
Example of user interface, one drug class. Underlined headings, e.g. Neuramidase inhibitors, are links to pop-up windows

Figure 1 shows the number of facts, relevant for the selection of appropriate drug class in the 246 studied diseases. Three algorithms were sufficient for the intended purpose. The simplest model is a list, containing the data, which imply that the patient should or should not receive a particular treatment. This model is adequate in situations where only one pharmacological treatment modality exists, such as influenza, where neuramidase inhibitors are the only drug class approved in Sweden, see figure 2. A 16 page summary of 35 pages of background documentation contained the following data items influencing drug therapy: four risk groups, time from start of symptom, two available neuramidases, one of which was approved also for use in children. Pharmaceutical properties regarding all approved drugs is available online (accessed from the program using 'deep linking'). In the actual program implementation information was added about current epidemiological state regarding influenza by linking to a government authority website, diagnostic criteria and some management aspects (in pop-up windows) (Fig. 2). 

**Figure 3:**
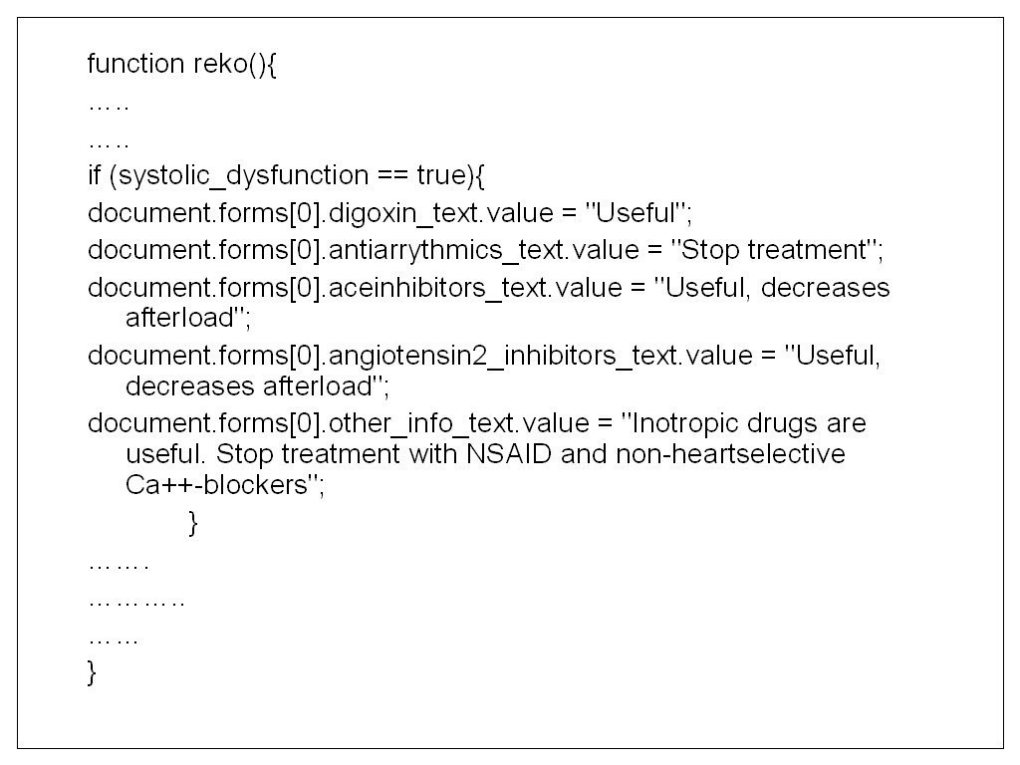
Example of a set of rules (verbose pseudocode)

**Figure 4:**
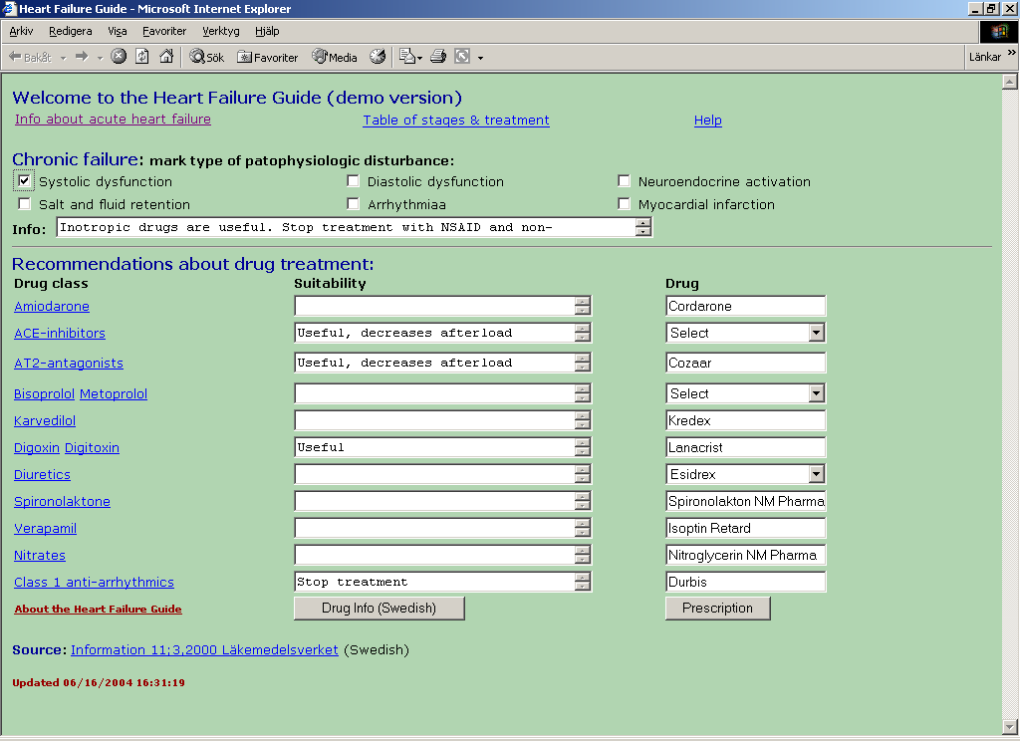
Example of user interface. Underlined headings are links to pop-up windows, check boxes refer to facts influencing selection of drugs.

In situations, where treatment can by provided using drugs from different pharmacological classes, a more elaborate model is required. An example is heart failure. A 25-page summary of 69 pages of background documentation described six physiological states and 11 available drug classes. This knowledge was modelled using an ordered set of it-then statements, see figure 3. The function containing these sets of ordered rules is activated every time the user enters some information on the screen. For an example of user interface, see figure 4. In the actual program implementation information was added about treatment in emergency situations and in different clinical stages of the disease (in pop-up windows) (Fig. 4). The algorithms mainly consist of sets of if-then statements, grouped in a single function, which is activated each time the user enters some information by checking a check box or radio button on the screen [20]. This information is equivalent to a fact list, and the if-then rules mimic a knowledge base. Thus in these aspects the algorithms are similar to conventional expert systems [21], but a separate inference engine was not needed. However, the if-then rules had to be ordered according to the output such that positive information, i.e. recommendations to use a particular drug, always was overwritten when factors were present that implied that this particular drug was contra-indicated or less suitable. Such sets of if-then statements were able to manage the identified information in all, but one instance. The exception was depression, where explicit knowledge regarding the relationships between patient characteristics and optimum choice of drug is lacking.

**Figure 5:**
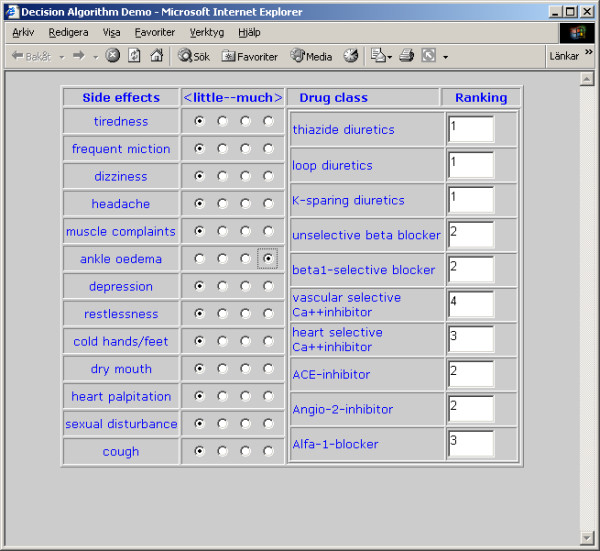
Output from a program for adjusting medication for high blood pressure according to the patient's side effects, in this case ankle edema

In some diseases the selection and dosage of drugs depend on the degree to which one or more particular factors are present. In such cases a specific program implementation of an established decision model based on fuzzy set theory [22] could be used. An example is the adjustment of medication according the patient's description of side effects in the treatment of high blood pressure [6,23], see figure 5. The data in this program were obtained from a study of 1013 treated and 125 untreated hypertensive patients [24]. Thirteen types of complaints were recorded and correlated to the ten available drug classes. The resulting matrix was analysed by four physicians, one professor of endocrinology, with special interest in hypertension, two clinical pharmacologists and one family physician, and modified according to scientific evidence and clinical experience. Clicking on radio buttons enters the type and degree of complaint and the program ranks the available drug classes according to the degree to which they can be assumed not to cause the patient's complaint(s). The algorithm is an implementation of a decision theory, based on fuzzy sets [22]. A verbal description of the implementation would be quite lengthy, but the workings of the algorithm and the code can be seen on the author's website [23].

The pharmaceutical information about all therapeutics drugs, approved in Sweden, is available on the Internet at a site managed by the association of pharmaceutical industries [19]. By linking to this site, the programs provide access to the relevant and continously updated drug information regarding all drugs, registered for use in the current disease. In addition, each program is linked to the appropriate guideline document, so that the user can verify the program's treatment advice.

## Discussion

The main findings in the current study are that simple algorithms can support drug therapy and that such algorithms can be easily implemented using standard Internet programming techniques. Problems in the implementation of information system designed to support actual care are discussed in a study by Bates et. al. [25]. The authors concluded that 'ten commandments' have to be taken into account. The most important factors for success were speed, anticipation of needs and good fit into the user's workflow. The programs developed in the current study easily meet these criteria, provided that the users have access to the Internet. However, it should be noted that the programs require no communication of patient-related information over the Internet, other than transmission of the prescription to the pharmacy, if such a system is implemented.

One disorder, depression, was identified for which relevant computer program support could not be constructed. Initially, the program model with the above-mentioned decision algorithm, based on fuzzy sets, was thought to be applicable, since the number of relevant facts is well within manageable range; four major classes of drugs, slightly more than ten diagnostic variables, some ten receptors and seven neurotransmitters. However, the information in the official consensus document [26], did not provide sufficient information, due to the lack of explicit relationships between the patient's characteristics, as described e.g. by diagnostic rating scales, and the pharmacological properties of the various types of antidepressants. Outcome studies regarding depression also give little information about which drug should be recommended for a particular patient. The exception is a recent report by Joyce et al [27], who found a markedly superior response to nortriptyline compared to fluoxetine in men above 40 years of age, and the reverse effect in women, aged 18 – 24 years.

Problems in pharmacotherapy related to the simultaneous occurrence of multiple disorders in the same patient are well studied and presented in the pharmacological information as contraindications or recommendations of "caution in patients with disease ...". This information is used as output from the programs in the current study.

The information in the analyzed text documents was not always sufficiently detailed and logically consistent to allow the construction of computer code. Additional information could usually be gathered from other sources, but such procedures may induce errors and should be avoided. Methods are currently available to support logical structure in text documents, e.g. the Guideline Elements Model [8]. This model relies on XML tagging, a standard for content description in documents intended for electronic processing. A formal structure would increase the usefulness of guidelines [24], since it would be possible to present them in the format of fast, easily available and "easy-to-use" programs, as in the recently published PresGuid system [11], which relies on XML tagging of text based guidelines for automatic conversion to computer programs. Experience will tell whether this approach will be more efficient than the simpler strategy of manual extraction of relevant data from the guideline document, combined with JavaScript coding, used in the current study.

The introduction of a new drug for treatment of a particular disease, or the results from new scientific studies may have considerable influence on guidelines. Yet only a few documents were changed during the study period and the lifespan of pharmacotherapeutic guidelines can be assumed to be 2–5 years. However, new information may appear unexpectedly, and a reasonably complete system for pharmacotherapy requires frequent updates. The approach taken in the current study, with the programs stored on a website as scripted HTML pages, facilitates the maintenance and update of the system and ensures that all users always have access to the latest version. An additional way to increase relevance over time is to link from the programs to recently published studies, e.g. by providing automatic searches in the PubMed database [28].

## Conclusion

In the current study based on guidelines regarding two hundred and forty-six diseases, three basic program models were found to be sufficient for computerized decision-support in pharmacotherapy. The programs could be developed using HTML and JavaScript, i.e. simple and widespread programming techniques. No specific development tools are needed although standard programming tools such as Macromedia Dreamweaver^® ^are helpful. The use of Internet implies that only one version of the programs is in use, that upgrading is very convenient, and that no interference occurs with existing systems. No confidential patient data has to be communicated and the programs are always available from any Internet connected computer.

In summary, algorithms for optimizing drug therapy can, with presumably rare exceptions, be developed for any disorder using standard Internet programming methods.

## Competing interests

None declared.

## Authors' contributions

PW conceived and designed the study, collected data, supervised data analysis, did most of the JavaScript and some of the HTML programming and contributed to the writing of the paper.

LM did most of the HTML and some of the JavaScript programming and contributed to the data analysis and the writing of the paper.

Both authors read and approved the final manuscript.

## Pre-publication history

The pre-publication history for this paper can be accessed here:
